# Transition Metal Dichalcogenides Nanoscrolls: Preparation and Applications

**DOI:** 10.3390/nano13172433

**Published:** 2023-08-27

**Authors:** Shilong Yu, Pinyi Wang, Huihui Ye, Hailun Tang, Siyuan Wang, Zhikang Wu, Chengjie Pei, Junhui Lu, Hai Li

**Affiliations:** Key Laboratory of Flexible Electronics (KLOFE) & Institute of Advanced Materials (IAM), Nanjing Tech University, Nanjing 211816, China

**Keywords:** TMDCs, nanosheet, nanoscroll, preparation, photodetection, hydrogen evolution reaction, gas sensing

## Abstract

Two-dimensional (2D) transition metal dichalcogenides (TMDCs) nanosheets have shown extensive applications due to their excellent physical and chemical properties. However, the low light absorption efficiency limits their application in optoelectronics. By rolling up 2D TMDCs nanosheets, the one-dimensional (1D) TMDCs nanoscrolls are formed with spiral tubular structure, tunable interlayer spacing, and opening ends. Due to the increased thickness of the scroll structure, the light absorption is enhanced. Meanwhile, the rapid electron transportation is confined along the 1D structure. Therefore, the TMDCs nanoscrolls show improved optoelectronic performance compared to 2D nanosheets. In addition, the high specific surface area and active edge site from the bending strain of the basal plane make them promising materials for catalytic reaction. Thus, the TMDCs nanoscrolls have attracted intensive attention in recent years. In this review, the structure of TMDCs nanoscrolls is first demonstrated and followed by various preparation methods of the TMDCs nanoscrolls. Afterwards, the applications of TMDCs nanoscrolls in the fields of photodetection, hydrogen evolution reaction, and gas sensing are discussed.

## 1. Introduction

As representative two-dimensional (2D) materials, the transition metal dichalcogenides (TMDCs) nanosheets have been successfully applied in the fields of photodetection [1,2,3,4,5,6], energy storage [7,8,9,10,11], catalysis [12,13,14,15,16], and so on. Although the monolayer TMDCs nanosheets have shown excellent optoelectronic performance, their low light absorption efficiency hinders the applications in photodetection [17], because of their ultrathin thickness.

A great deal of effort has been developed to improve the light absorption of TMDCs nanosheets, such as plasma treatment, formation of van der Waals heterojunction, utilization of plasmonic effect, integration of quantum dots [18,19,20,21,22], etc. Recently, rolling up the monolayer TMDCs nanosheet to form the TMDCs nanoscroll (TMDCs-NS) has been reported as a promising method to improve their optoelectronic performance [19]. With the aid of volatile organic solvents or alkaline solution [19,23,24,25,26,27], the monolayer TMDCs nanosheets can be transformed into one-dimensional (1D) nanoscrolls with tubular and spiral structures [23,28,29]. The as-obtained TMDCs nanoscroll (TMDCs-NS) showed enhanced light absorption due to the increased cross-section. In addition, the TMDCs-NS also inherits the excellent properties from the monolayer TMDCs nanosheet [21,30,31,32,33,34,35]. Furthermore, the curved structure of the nanoscroll exhibits strain, which can modulate the band gap of TMDCs nanosheet [33]. Moreover, its scrolled structure has tunable interlayer space with open ends, in which other functional nanomaterials can be encapsulated [35,36]. Therefore, the TMDCs-NS have attracted great attention in optoelectronics in recent years.

In this review, we introduce the structure, fabrication, and applications of TMDCs-NS. Firstly, the structure of nanoscrolls is demonstrated. Secondly, the preparation methods of nanoscrolls are discussed in detail. We then present the applications of TMDCs-NS in the photodetection, sensing, and hydrogen evolution reaction.

## 2. Structure of TMDCs-NS

**Figure 1:**
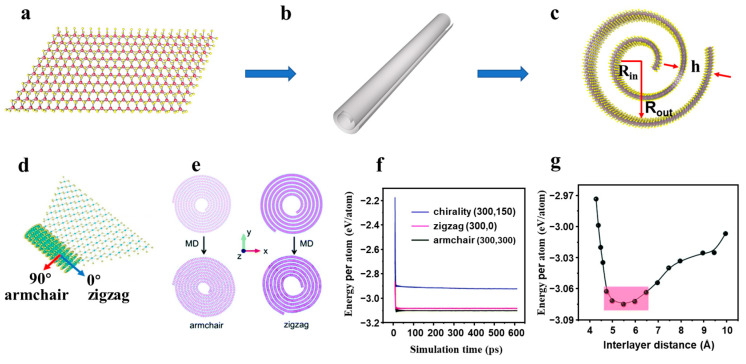
(**a**). Monolayer MoS_2_ nanosheet. (**b**) Schematic structure of the TMDCs nanoscroll with open ends and side edges. (**c**) The MoS_2_ nanoscroll with inter-core radius R_in_, in outer radius R_out_ and interlayer spacing h. (**d**) The formation of the MoS_2_ nanoscrolls are always along the armchair direction (Mo–S bond direction) [56]. (**e**) The MoS_2_ sheets of the same size roll through the armchair (left) and zigzag (right) orientation molecular dynamics (MD) simulations before (upper) and after (lower) structural relaxation [53]. (**f**) The relationship between unit atomic energy and simulation time of MoS_2_ nanoscrolls with armchair, zigzag, and chiral orientation [53]. (**g**) The atomic energy is a function of MoS_2_ nanoscroll spacing. The red area indicates the energy-favorable interlayer distance [53].

Different from other 1D nanomaterials, the nanoscrolls have spiral tubular structure with weak van der Waals (vdW) interaction between adjacent layers, which are transformed from 2D nanosheet, exhibiting open ends and side edges without fusion as shown in Figure 1b, To date, various 2D nanosheets can be transformed into nanoscrolls, such as graphene, TMDCs, black phosphorus and h-BN [37,38,39,40,41,42]. The graphene nanoscroll was discovered in the arc discharge experiment of graphite electrode [43], and confirmed by transmission electron microscopy (TEM) [44]. The large-scale preparation of graphene nanoscroll was proposed in 2003 and then received much attention [45,46].

Similar to graphene nanoscroll, the TMDCs-NS is also composed of monolayer TMDCs nanosheets scrolled up into Archimedean helical structure (Figure 1c) [37,47]. These TMDCs-NS have unique physical and chemical properties due to their nanoscale dimensions and high surface area-to-volume ratio, which make them useful in a variety of applications, such as energy storage, catalysis, and optoelectronics [19,27,48,49,50,51,52]. 

The structure of TMDCs-NS is illustrated by taking MoS_2_ nanoscroll as an example. By scrolling a monolayer MoS_2_ nanosheet (Figure 1a), the MoS_2_ nanoscroll is formed with an inner layer radius of R_in_, an outer layer radius of R_out_, and a interlayer spacing of h, as shown in Figure 1c [53,54,55]. The scrolling direction is usually along the armchair or zigzag orientation of the MoS_2_ nanosheet (Figure 1d) [53]. A molecular dynamics (MD) simulation was performed to understand the scrolling direction of MoS_2_ at the molecular level (Figure 1e). The results indicate that the energy per atom of nanoscrolls along the armchair orientation is lower than those of nanoscrolls through the zigzag and chiral orientations (Figure 1f). As a consequence, the armchair orientation (Mo-S bond direction) is the dominant trend to roll up the MoS_2_ nanosheet into MoS_2_ nanoscrolls [56]. The spacing between adjacent layers of MoS_2_ nanoscroll also plays an important role in determining its stability. As the interlayer spacing increases from 4 to 5.5 Å, the energy of each atom decreases quickly (Figure 1g). While it increases continuously as the interlayer spacing increases from 5.5 to 10 Å. The MoS_2_ nanoscroll is in an energy-favorable state when the interlayer spacing is in the range of 4.7~6.5 Å [47,53].

## 3. Preparation of TMDCs Nanoscrolls

In recent years, many TMDCs nanosheets have been reported to form TMDCs-NS, including MoS_2_, WS_2_, MoSe_2_, MoS_2_/WS_2_, etc. [3,9,17,18,19,20,29,33,51,52,53,57,58,59,60,61,62,63,64,65,66,67,68]. Several methods have been successfully developed to prepare TMDCs-NS, such as strain-induced scrolling [17], argon plasma-assisted scrolling [18], supercritical fluid-assisted scrolling [68,69], volatile organic solvent-induced scrolling [23,24,25,33,70], alkaline droplet assisted scrolling [19,21,26,27,35], and vortex flow device (VFD) induced continuous flow [50]. 

Theoretical investigation indicates that there is an energy barrier between the nanosheet and nanoscroll [71,72,73]. A driving activation energy is required to initialize the scrolling automatically [74]. By scrolling or folding the nanosheet into a nanoscroll, the lowest energy form is presented [74]. The barrier can be overcome with the help of external force in liquid or in air, where the TMDCs-NS is formed. 

### 3.1. Fabrication of TMDCs Nanoscrolls in Liquid

#### 3.1.1. Solvent Evaporation to Make Nanoscrolls

**Figure 2:**
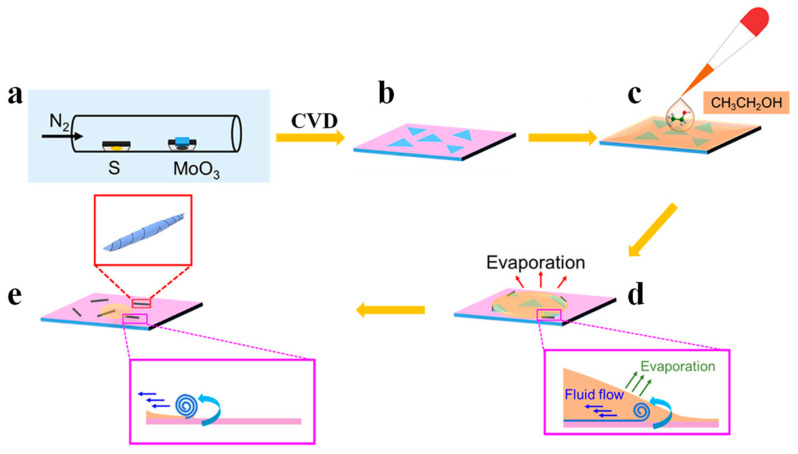
Volatile organic solvent-assisted fabrication of MoS_2_ nanoscroll [23]. (**a**,**b**) CVD growth of monolayer MoS_2_ nanosheets. (**c**) A drop of ethanol is deposited on MoS_2_ nanosheets. (**d**) The edges of MoS_2_ nanosheets are rolled up during the evaporation of ethanol. (**e**) MoS_2_ nanoscrolls were formed.

Many organic solvents are liquid at ambient conditions with large volatility, which can be used to assist or induce the scrolling of TMDCs nanosheets [23,33,35,75,76,77], such as acetone, ethanol, and isopropanol [24,78,79,80]. In 2018, we proposed the preparation of MoS_2_ nanoscroll by dropping an ethanol or acetone droplet on monolayer MoS_2_ nanosheet [23], as shown in Figure 2. Monolayer MoS_2_ nanosheets were first obtained by chemical vapor deposition (CVD) on SiO_2_/Si (Figure 2a,b), and a drop of ethanol was deposited on the MoS_2_ nanosheets (Figure 2c). Due to its low surface tension, the ethanol can wet MoS_2_ and SiO_2_/Si substrates easily. During the evaporation process, a thin ethanol layer is formed near the contact line (Figure 2d). With the rapid evaporation of ethanol, a temperature gradient is generated near the contact line, creating a surface tension gradient to induce fluid flow. Such kind of fluid flow could roll up the edge of MoS_2_. As the contact line moves, the MoS_2_ nanosheet continues to be rolled up until a complete MoS_2_ nanoscroll is formed (Figure 2e) [23]. 

**Figure 3:**
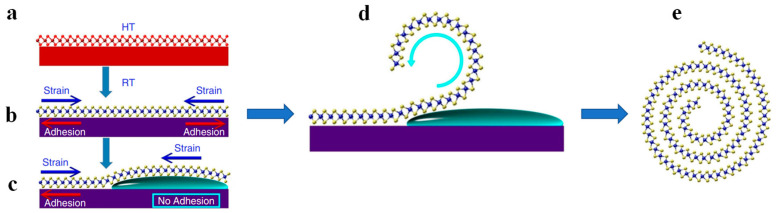
The mixture of ethanol and water-assisted fabrication of MoS_2_ nanoscroll [24]. (**a**) CVD growth of MoS_2_ nanosheet. (**b**) Strain-balanced MoS_2_ on substrate. (**c**) After ethanol intercalation, the strain-adhesion balance between MoS_2_ and substrate is broken. (**d**) Rolling up the edge of the MoS_2_ nanosheet. (**e**) The as-prepared MoS_2_ nanoscroll.

By dropping the mixture of ethanol and water on CVD-grown monolayer MoS_2_ nanosheet, Jian Zheng et al. also successfully prepared MoS_2_ nanoscrolls, as shown in Figure 3 [24]. Large-area monolayer MoS_2_ nanosheets were obtained firstly by CVD (Figure 3a). MoS_2_ nanoscrolls were then fabricated in a short time in a mixture solution of ethanol and water with a volume ratio of 2:1 (ethanol:water = 2:1).

Due to the high temperature-causing mismatch between the MoS_2_ nanosheet and substrate during CVD growth (Figure 3a), a strain equilibrium is balanced between them when the temperature decreases to room temperature (Figure 3b). When the mixture solution is dropped onto the MoS_2_ nanosheet, the ethanol intercalates between the MoS_2_ and substrate, and the upper part of MoS_2_ is detached from the substrate. In this case, the strain balance is broken (Figure 3c), and the released portion curls into a roll (Figure 3d). Due to the adhesion from the substrate, the left portion remains intact [24]. With the evaporation of ethanol, the surface tension at the air-solvent-MoS_2_ interface is greater than that between MoS_2_ and the substrate. As a result, the strain-adhesion balance is broken continuously, generating MoS_2_ nanoscroll finally (Figure 3e). 

#### 3.1.2. Alkaline Droplet-Assisted Fabrication of Nanoscrolls

**Figure 4:**
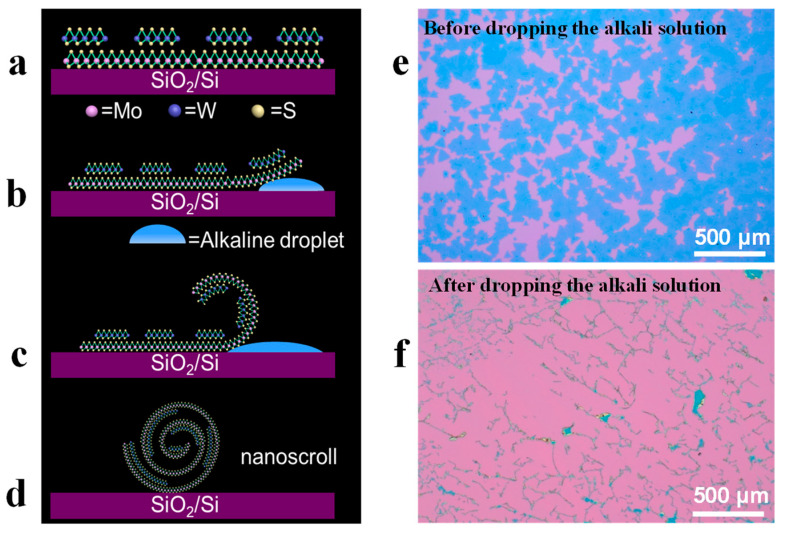
Alkaline droplet-assisted fabrication of nanoscrolls [19]. (**a**) CVD-grown MoS_2_/WS_2_ heterostructures nanosheet on SiO_2_/Si substrate. (**b**) Etching the SiO_2_ layer beneath the nanosheet by dropping an alkaline solution. (**c**) The edge of the WS_2_/MoS_2_ nanosheet is rolled up due to the elimination of strong adhesion from the substrate. (**d**) The as-formed WS_2_/MoS_2_ heterojunction nanoscroll. (**e**,**f**) Show the comparison before and after dropping the alkali solution on the MoS_2_/WS_2_ nanosheets.

For bilayer and multilayer nanosheets, it is difficult to roll them up effectively due to the strong adhesion force from the substrate. Since TMDCs nanosheets are usually grown on SiO_2_/Si substrate, etching the SiO_2_ layer beneath them could eliminate the strong adhesion from the substrate. Therefore, alkaline solution has been used to etch the SiO_2_ layer, and thus break the adhesion energy between the nanosheet and substrate (Figure 4). As a result, the strain equilibrium is broken, rolling up the TMDCs nanosheet from edges to form spiral nanoscrolls [19,51]. 

The materials for fabricating nanoscrolls are derived from 2D nanosheets produced by mechanical stripping [78] or CVD. The experiments were dominated by CVD-prepared nanosheets, but the nanosheets prepared by CVD in most cases are accompanied by the production of defects, homojunctions, and heterojunctions [81]. Therefore, we successfully fabricated nanoscrolls using CVD-prepared heterojunction nanosheets. In 2020, we proposed a method to scroll the MoS_2_/WS_2_ heterostructures nanosheet by using an alkaline solution. Firstly, large-area MoS_2_/WS_2_ heterostructure nanosheets were grown on SiO_2_/Si substrate by CVD (Figure 4a) [19,81]. Afterwards, 50 μL of alkaline aqueous solution (0.1 M KOH or NaHCO_3_) was dropped on the nanosheets. The SiO_2_ layer was etched by the alkaline solution, allowing penetration of the alkaline solution into the interface of the nanosheet and SiO_2_/Si substrate (Figure 4b), which could further etch the SiO_2_ layer to release the edge of the nanosheet. To decrease the energy form, the released edge of the nanosheet tends to be scrolled (Figure 4c). With the further etching of the SiO_2_ layer, more portions of the nanosheet were released and continuously scrolled till forming nanoscroll (Figure 4c,d) [19]. The as-obtained nanoscroll was then rinsed with deionized (DI) water and dried with N_2_ for characterization and device fabrication (Figure 4d). Furthermore MoS_2_/WS_2_ heterostructures nanosheet, we found that the silver nanoparticles decorated monolayer MoS_2_ and WS_2_ nanosheets could also be rolled up effectively by using an alkaline solution [35]. 

Duan et al. found that 80% of the bilayer and trilayer TMDCs heterostructure nanosheets could be transformed into 1D nanoscrolls by using a mixture of ethanol and water. However, many scrolls are incompletely rolled up with tightly pinned edges, indicating the mixed solvent does not work well to completely delaminate the edges of a thick 2D nanosheet. The yield of the nanoscroll increased to 90% by adding 5% ammonia into the mixed solvent, with 60% of them showing a closely stacked scroll structure. The result indicated that the etching of the SiO_2_ layer by alkaline solution played an important role in peeling off the 2D nanosheet from the substrate even the existence of strong edge-substrate interaction [21,24]. 

Similarly, alkaline solution was also employed to roll up PbI_2_/MoS_2_ and BaTiO_3_/MoS_2_ nanosheets into complete nanoscroll, further confirming the importance of alkaline solution in preparation of TMDCs-NS [3,27,51]. 

#### 3.1.3. Fabrication of TMDCs Nanoscrolls by Dragging Water Droplets

**Figure 5:**
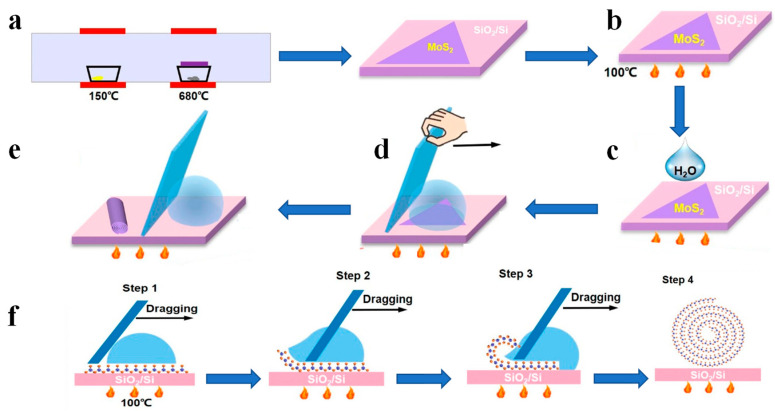
Fabrication of TMDCs nanoscrolls by dragging water droplets at 100 °C [52]. (**a**) Monolayer MoS_2_ nanosheet was grown by CVD at 680 °C. (**b**) Heat the substrate and MoS_2_ nanosheets for 10 min. (**c**) A drop of H_2_O on the MoS_2_ film. (**d**) The H_2_O droplet was dragged across the MoS_2_ nanosheet on the SiO_2_/Si substrate by a coverslip at 3 mm/s^−1^; (**e**) MoS_2_ nanoscroll was formed after removing the H_2_O droplet; (**f**) Schematic diagram of the formation of the MoS_2_-NS.

Due to the low surface tension of organic solvent, it is easily adsorbed on TMDCs nanosheets. Thus, it is inevitable to trap organic solvent in the TMDCs-NS during the scrolling process. It is known that the adsorbed organic solvent could greatly influence the properties and device performance of TMDCs nanosheets. Therefore, it is desirable to fabricate TMDCs-NS without using organic solvent. Recently, we reported an organic solvent-free method to fabricate tightly-packed TMDCs-NS [52]. Firstly, CVD-grown monolayer MoS_2_ nanosheets were heated at 100 °C, as shown in Figure 5a,b. After 3 µL of deionized (DI) water droplet was dropped on the MoS_2_ nanosheets (Figure 5c), The coverslips were dragged from one end to the other at a speed of 3 mm/s (Figure 5d). Afterwards, large scale closely packed MoS_2_ nanoscrolls were fabricated (Figure 5e). 

The detailed mechanism for the formation of nanoscrolls could be explained as follows, as shown in Figure 5f. When the monolayer MoS_2_ nanosheets on SiO_2_/Si substrate were heated at 100 °C, the adhesion force between the nanosheets and substrate was weakened. Therefore, with the movement of water droplets, the scrolling occurs first at the edges of MoS_2_ nanosheets. Due to the hydrophobicity of MoS_2_ nanosheets, the low friction between water and MoS_2_ and the high surface tension of water contribute to the following scrolling of MoS_2_ nanosheets. More importantly, due to the hydrophobicity of MoS_2_, the water molecules were difficult to trap in the nanoscrolls, obtaining solvent-free and closely packed nanoscrolls [52]. 

#### 3.1.4. Amine-Functional Amphiphilic Molecule Assisted Fabrication of TMDCs Nanoscrolls

**Figure 6:**
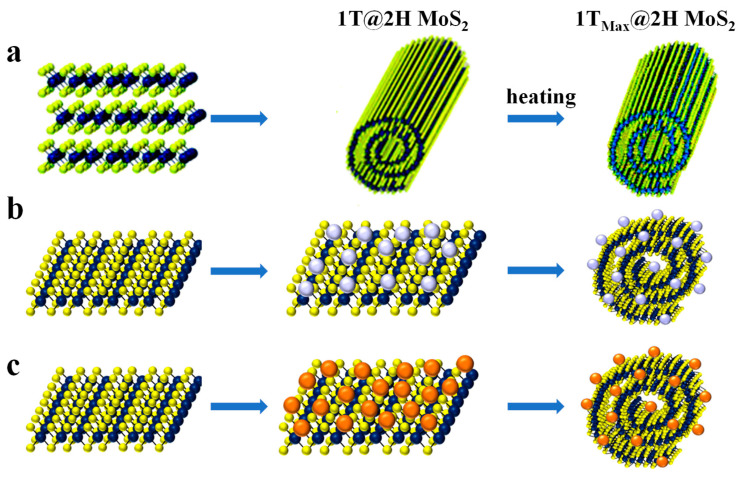
Amine-functional amphiphilic molecule assisted fabrication of TMDCs nanoscrolls. (**a**) Scheme of Amine-functional amphiphilic molecule assisted fabrication of 1T@2H MoS_2_ nanoscrolls [49]. (**b**,**c**) Scheme of Amine-functional amphiphilic molecule assisted fabrication of MoS_2_-Ag and MoS_2_-Au nanoscrolls [83].

The involvement of amphiphilic molecules in the preparation of MoS_2_, MoSe_2_, and MoTe_2_ nanoscrolls has also been investigated [48,49,82,83]. By mixing N-(2-aminoethyl)-3α-hydroxy-5β-cholan-24-amide (LCA) and exfoliated TMDCs nanosheets in orthodichlorobenzene (ODCB) for 24 h at room temperature, TMDCs-NS was obtained in large scale (Figure 6a). The formation of MoS_2_ nanoscrolls with the help of LCA could be explained as follows. Firstly, the LCA molecules were self-assembled into fibers in ODCB. The amine group of LCA fiber has a stronger interaction with the edges of TMDCs nanosheets than the entire plane. Thus, the edges start scrolling around the LCA fibers. With the gradual self-assembly of LCA fiber, the interaction of fiber with the edges of TMDCs nanosheets is enhanced. In this case, the original equilibrium state of TMDCs nanosheets is broken and the nanosheet scrolls spontaneously. Therefore, TMDCs-NS was formed. By using this method, MoS_2_ nanoscrolls, MoS_2_-Ag nanoparticles nanoscrolls, and MoS_2_-Au nanoparticles nanoscrolls have been successfully prepared [49,83]. 

#### 3.1.5. Supercritical Fluid-Assisted Fabrication of Nanoscrolls

**Figure 7:**
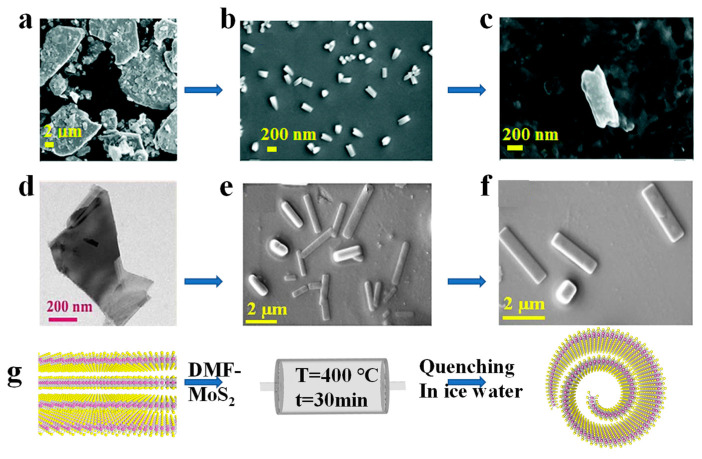
Supercritical fluid processing-assisted fabrication of TMDCs nanoscrolls. FE-SEM images of (**a**) bulk MoS_2_ flake and (**b**,**c**) supercritical fluid-prepared MoS_2_ nanoscrolls [88]. FE-SEM images of (**d**) bulk WS_2_ flake and (**e**,**f**) supercritical fluid prepared WS_2_ nanoscrolls; (**g**) Schematic diagram of supercritical fluid processing-assisted preparation of TMDCs nanoscrolls [68].

Supercritical fluids (SCFs) are fluids with much more space and are highly compressible than ordinary fluids above their critical temperatures and pressure [84,85]. By controlling the temperature or pressure, the density and solvent strength of SCFs can be tuned from gas-like to solid-like [49,83,86,87]. SCF has unique properties such as gas diffusivity, liquid solubility, low interfacial tension, excellent surface wettability, and high diffusion coefficient [88,89,90]. Thus, SCFs processing has been used as a promising and effective route to exfoliate layered materials into 2D nanosheets, such as graphene, BN, and MoS_2_ due to their simplicity, rapidity, and short reaction time [68,69,88,91]. It has been reported that the as-exfoliated 2D nanosheets can be rolled up into nanoscrolls in order to minimize their surface energy. Therefore, MoS_2_ and WS_2_ nanoscrolls can be formed by using SCF processing of MoS_2_ nanosheets in 30 min, as shown in Figure 7a–g [68,88]. The X-ray diffraction (XRD) patterns of the MoS_2_ nanoscrolls clearly show that the surfaces of the MoS_2_ nanosheets are not oxidized and are free of impurities [68]. Meanwhile, the lattice structure of the SCF-prepared MoS_2_ is essentially unchanged, making this method a convenient and efficient way to prepare nanoscrolls.

#### 3.1.6. Shear Force Assisted Fabrication of Nanoscrolls

**Figure 8:**
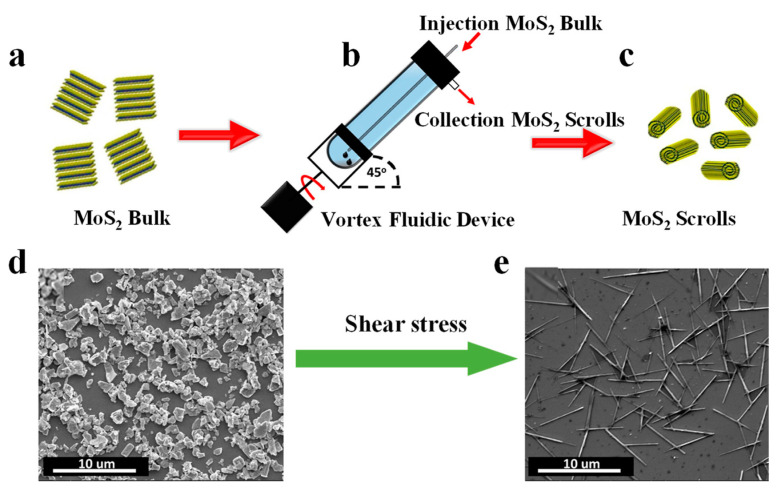
Shear force-assisted fabrication of nanoscrolls [50]. (**a**–**c**) Schematic illustration of the fabrication of MoS_2_ scrolls in VFD. SEM images of the (**d**) MoS_2_ bulk material and (**e**) as-prepared MoS_2_ scrolls in VFD.

By using a vortex flow device (VFD), MoS_2_ nanosheets have been successfully transformed into nanoscrolls under continuous flow [50]. In a tilted quartz tube with rapid rotation, a dynamic thin film was generated in VFD, providing mechano-energy as high shear stress during intense micro-mixing. Therefore, the MoS_2_ nanosheets were firstly exfoliated from bulk material due to the strong shear stress. Simultaneously, the as-exfoliated MoS_2_ nanosheets were rolled up in-situ to form scrolls with high yield (Figure 8a,b). At low-speed rotation (4000 rpm), the shear stress is mainly governed by the Typhoon-like toroidal flow, generating centrifugal forces on the tube wall. As a result, the MoS_2_ nanosheets were exfoliated first and then scrolled as the toroidal flow moved upwards (Figure 8c,d) [50,92,93]. As the rotation speed increased to 8000 rpm, the dominated double-helical twisted Faraday wave vortex flow cannot curl and roll up MoS_2_ nanosheets effectively. The morphology of MoS_2_ nanostructure can be changed from lamellae to scroll, by controlling the solvent selection, concentration of bulk material, and the processing parameters of VFD, including rotation speed and rotation angle. The VFD has been widely used to synthesize nanoscrolls from 2D nanosheets, such as graphite, graphene oxide, and hexagonal boron nitride [92,93,94]. 

### 3.2. Fabrication of TMDCs Nanoscrolls in Air

#### 3.2.1. Plasma-Assisted Fabrication of MoS_2_ Nanoscrolls

**Figure 9:**
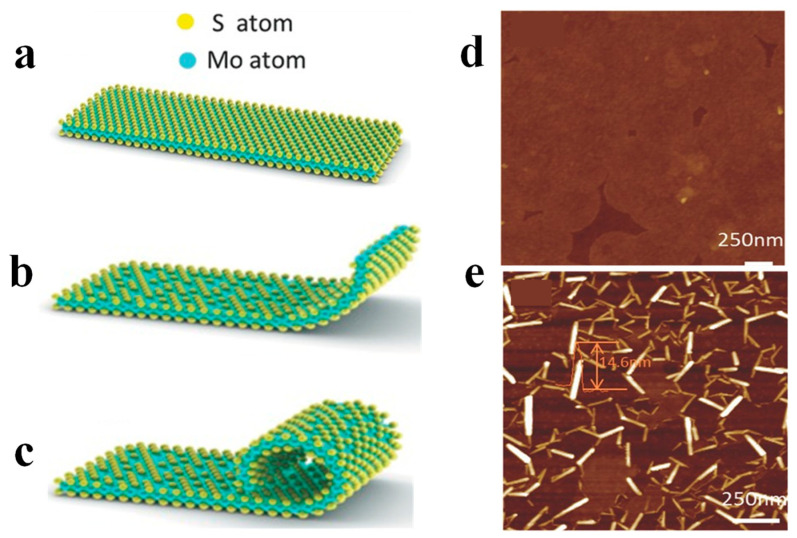
Ar plasma-assisted fabrication of MoS_2_ nanoscrolls [18]. Schematic structures of (**a**) monolayer, (**b**) edge distortion, and (**c**) scrolled edge of MoS_2_ nanosheet under Ar plasma treatment. AFM images of (**d**) monolayer MoS_2_ nanosheet and (**e**) as-obtained MoS_2_ nanoscrolls.

In 2016, Zhang et al. proposed the preparation of MoS_2_ nanoscrolls by treating CVD-grown monolayer MoS_2_ nanosheets in a weak Ar plasma environment, as shown in Figure 9 [18]. Upon plasma bombardment, the top layer sulfur atoms of the MoS_2_ nanosheets are partially removed as the kinetic energy of Ar^+^ is larger than the binding energy of the Mo-S bond. As a result, the MoS_2_ lattice is disrupted and unsaturated dangling bonds are left, leading to out-of-plane strain. Such kind of strain induces out-of-plane distortion, which rolls up the edge of the MoS_2_ nanosheet to form nanoscrolls [18,58]. The optimum power for fabricating nanoscrolls was 25 W. If the power was too strong, short nanoscrolls were obtained. While a longer time was needed to trigger the scrolling when the power was too weak. When the adjacent edges of MoS_2_ nanosheets are not parallel, a kink will be formed, preventing the formation of a long scroll [18]. In addition, WS_2_ and WSe_2_ nanoscrolls were also prepared by treating the monolayer WS_2_ and WSe_2_ nanosheets in Ar plasma.

#### 3.2.2. Strain-Assisted Fabrication of TMDCs Nanoscrolls

**Figure 10:**
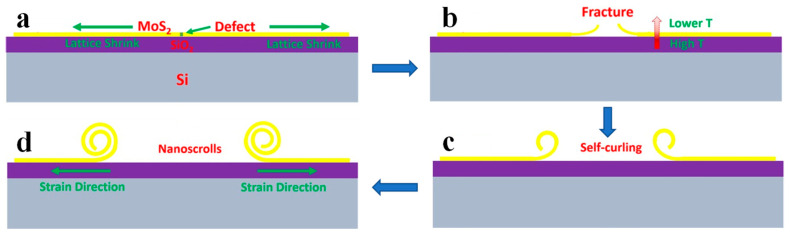
Schematic diagram of nanoscroll formed by thermal strain [96]. (**a**) Monolayer MoS_2_ nanosheets were prepared on SiO_2_/Si substrate by CVD. (**b**) S vacancy acts as crack nuclei due to the thermal strain upon quenching. (**c**) Self-curling at the fractures of the MoS_2_ layer because of the temperature difference between the MoS_2_ and SiO_2_ layer. (**d**) The MoS_2_ nanoscroll is formed by thermal strain.

Because of the different thermal expansion coefficients of the MoS_2_ and SiO_2_, there are thermal strain gradients between the interface of CVD-grown MoS_2_ nanosheets and SiO_2_/Si substrate [58,75,95]. Upon quenching, the orientation-specific fractures are formed on CVD-grown MoS_2_ nanosheets due to the existing S vacancies (Figure 10a,b). Since the cooling rate of the top MoS_2_ layer is greater than the bottom SiO_2_ layer, strong lattice contraction of the MoS_2_ layer is observed due to the temperature difference, which induces self-curling at the fractures of the MoS_2_ layer (Figure 10c). Afterwards, the curled edge continues to form a scroll in order to minimize the surface energy (Figure 10d).

Table 1 summarizes the preparation methods of TMDCs nanoscrolls to show the advantages and disadvantages in detail. To date, the TMDCs nanoscrolls have been prepared mainly from CVD-grown monolayer nanosheets or film in large-area. It is known that large-area TMDCs nanosheets can also be obtained by using molecular beam epitaxial (MBE) method and mechanical exfoliation (ME). Therefore, TMDCs nanosheets prepared by the MBE or ME method provide an alternative way for fabricating TMDCs nanoscrolls. Currently, it is difficult to control the geometry of nanoscrolls by using organic or alkaline droplets to directly treat CVD-grown large-area monolayer TMDCs films. In this case, TMDCs nanoscrolls with lengths of several tens to hundreds of micrometers were obtained in random orientation. By using a focused ion beam (FIB), the large-area monolayer TMDCs film can be shaped as long parallel ribbons with controlled width and direction. After dropping the mixture of ethanol and water on these ribbons, long straight TMDCs nanoscrolls can be obtained in array form [24]. The diameter of TMDCs nanoscroll could be tuned by controlling the width of the original ribbon. By using FIB to cut the long TMDCs nanoscrolls, the TMDCs nanoscrolls were patterned into arrays with controlled lengths and locations. By etching the large-area monolayer TMDCs films along the crystalline orientation, TMDCs nanoscrolls arrays with controlled chirality could also be prepared.

## 4. Applications of TMDCs-NS

### 4.1. Photodetector Based on TMDCs-NS

**Figure 11:**
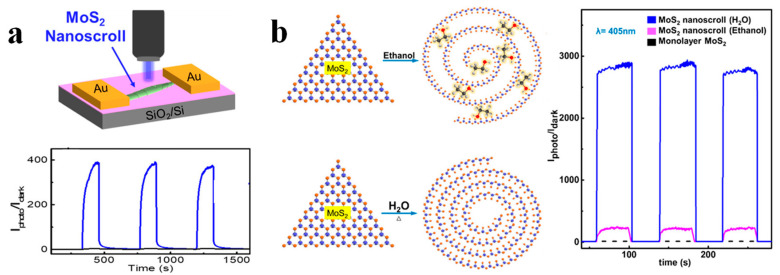
Photodetection performance of TMDCs-NS based device. (**a**) Top panel: Scheme of photodetector based on MoS_2_ nanoscroll. Bottom panel: PDR plot of photodetectors based on MoS_2_ nanosheet and nanoscroll under 405 nm laser [23]. (**b**) The scheme of nanoscrolls is made by dropping ethanol and water droplets, respectively. PDR plot of corresponding photodetectors under 405 nm laser [52].

Compared to monolayer TMDCs nanosheet, the TMDCs-NS shows much better light absorption efficiency due to its increased thickness of spirally scrolled structure. Thus, the TMDCs-NS should exhibit excellent optoelectronic performance. Recently, the photodetection performance of TMDCs-NS has been investigated [51,98]. Photosensitivity, described by the ratio of photocurrent to dark current (PDR), is one of the most important parameters to evaluate the performance of a photodetector [19,23,35]. Figure 11a shows the PDRs of MoS_2_ nanosheet and nanoscroll-based photodetectors under blue light irradiation with a bias voltage of 0.1 V. The PDR of a nanoscroll-based photodetector is about 400, which is about 100 times higher than that of a nanosheet-based one [23]. In addition, the response and recovery time of a nanoscroll-based photodetector is less than the nanosheet-based photodetector. Similar enhanced PDR was also observed in the MoSe_2_ nanoscroll-based device [17]. These results indicate that the TMDCs-NS shows much better photodetection performance than the TMDCs nanosheet, which should be attributed to the enhanced light absorption and rapid electron transportation along the 1D structure [24,52]. The photodetection enhancement of TMDCs nanoscrolls could be explained in detail as follows. Firstly, the increased thickness of TMDCs nanoscroll increases the light absorption as the light permeates each layer of the nanoscroll. Although each layer shows low light absorption, the total light absorption of the nanoscroll has increased. Therefore, increased photocurrent is obtained in nanoscroll. In addition, the one-dimensional structure of the nanoscroll confines the electron transportation along the axis direction, and thus rapid electron movement is realized in the nanoscroll compared to that in a 2D nanosheet. Moreover, due to the large surface-to-volume ratio of the MoS_2_ nanosheet, the adsorbates, such as O_2_ and H_2_O molecules, greatly reduce the photoresponse of the MoS_2_ nanosheet-based device. The MoS_2_ nanoscroll has a much smaller surface-to-volume ratio than the nanosheet, which can decrease the influence of adsorbates on the photoresponse. 

Compared to the MoS_2_ nanoscroll prepared by ethanol droplet (MoS_2_ NS-ethanol), the MoS_2_ nanoscroll prepared by water droplet (MoS_2_ NS-water) shows higher PDR (Figure 11b) [52]. The ethanol molecules trapped in MoS_2_ NS-ethanol reduce the light absorption and hinder the interlayer transport of photogenerated carriers, and thus decrease the photoresponse. In addition, the ethanol can donate an electron to MoS_2_ and thus increase the dark current of MoS_2_ NS-ethanol, which in turn decreases the PDR. Moreover, the MoS_2_ NS-water shows a slightly higher photocurrent than MoS_2_ NS-ethanol. 

### 4.2. Photodetector Based on TMDCs-NS Composite

**Figure 12:**
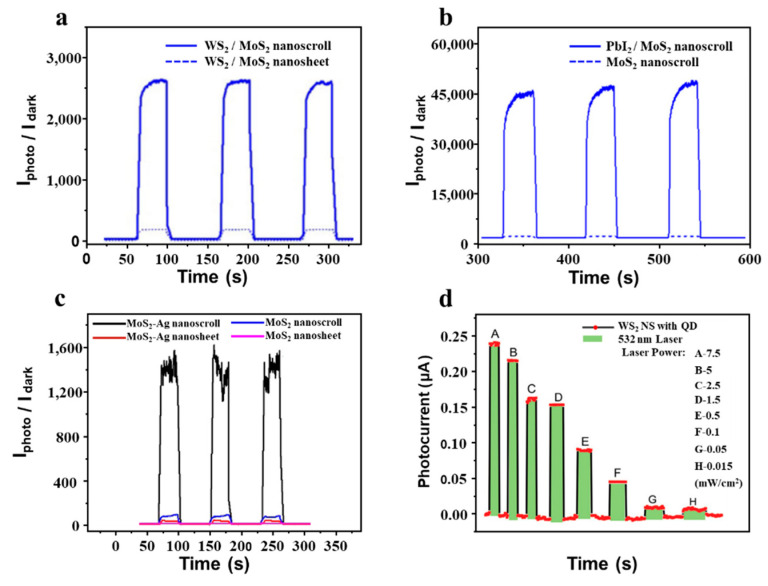
Photodetector based on TMDCs-NS composite. (**a**) Plots of the PDRs of photodetectors based on WS_2_/MoS_2_ nanosheet and nanoscroll under blue laser [19]. (**b**) PDRs plots of photodetectors based on MoS_2_ nanoscrolls and PbI_2_/MoS_2_ nanoscrolls under 405 nm lasers [51]. (**c**) Plots of the PDRs of the MoS_2_ nanosheet, MoS_2_ nanoscroll, MoS_2_-Ag nanosheet, and MoS_2_-Ag NS under a 633 nm laser [35]. (**d**) Photocurrent variations in hybridized WS_2_ nanoscroll photodetectors under different power densities of a 532 nm laser [79].

In bilayer WS_2_/MoS_2_ heterostructure, the carriers can be transferred from MoS_2_ to WS_2_ within 50 fs under illumination, indicating the important role of interface. However, there is only one interface in the bilayer WS_2_/MoS_2_ heterostructure. The photoresponse performance of WS_2_/MoS_2_ heterostructure could be further improved if multiple interfaces can be established. By scrolling the bilayer WS_2_/MoS_2_ heterostructure into WS_2_/MoS_2_ heterostructure nanoscroll, multiple hetero-interfaces are formed, which could show better photoresponse than the bilayer WS_2_/MoS_2_ heterostructure with one hetero-interface. After the bilayer WS_2_/MoS_2_ heterostructure was grown by CVD, the alkaline droplet was dropped on it to roll up the bilayer heterostructure into a nanoscroll [19]. As shown in Figure 12a, the PDR of bilayer WS_2_/MoS_2_ heterostructure-based photodetector is ~180 under a blue laser, which is much higher than that of monolayer MoS_2_ or WS_2_ nanosheets. The PDR of WS_2_/MoS_2_ heterostructure nanoscroll-based photodetector is 2700, about an order of magnitude higher than that of bilayer WS_2_/MoS_2_ heterostructure-based photodetector.

By encapsulating photoactive PbI_2_ nanocrystals into MoS_2_ nanoscroll, the PDR of MoS_2_ nanoscroll can be enhanced by two orders of magnitude (Figure 12b) [51]. Similarly, the PDRs of MoS_2_ and WS_2_ nanoscrolls also increased by two orders of magnitude after Ag nanoparticles were trapped in a nanoscroll (Figure 12c) [35]. Moreover, the photoresponsivity of the MoS_2_ nanoscroll was also enhanced by about two orders of magnitude when BaTiO_3_ nanoparticles were encapsulated into it [27]. By doping the WS_2_ nanoscroll with CdSe–ZnS quantum dots, the photosensitivity can be enhanced 3000-fold (Figure 12d) [79]. Compared to single TMDCs nanoscrolls, the nanoscroll composite shows excellent photodetection performance [24,70], indicating it could be a promising candidate for high-performance optoelectronics.

### 4.3. Hydrogen Evolution Reaction

The conductivity and effective active site of the catalyst are two key factors in improving the hydrogen precipitation reaction (HER) [99,100,101]. TMDCs are considered promising candidates after noble metals for catalytic hydrogen precipitation due to their good electrical conductivity and abundant active edges [66,102,103]. The introduction of a small amount of MoSx greatly enhanced the HER activity of NbS_2_ nanoflakes [100]. The theoretical calculation indicates that Mo edge sites are identified as the catalytically active site for HER [104,105]. 

**Figure 13:**
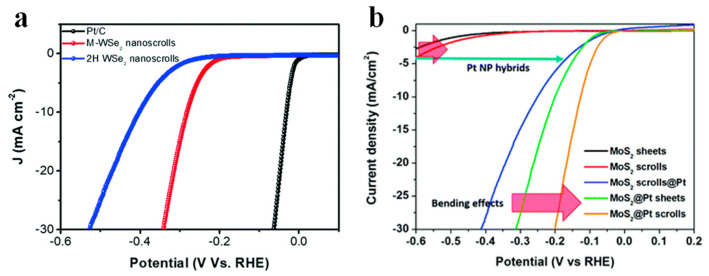
Hydrogen evolution reaction (HER) based on TMDCs-NS. (**a**) HER polarization curves for M-WSe_2_ nanoscrolls, 2H-WSe_2_ nanoscrolls, and the commercial Pt/C [103]. (**b**) Polarization curves for Pt electrode, MoS_2_ sheet, MoS_2_ nanoscroll, MoS_2_ nanoscrolls@Pt, MoS_2_@Pt sheet, and MoS_2_@Pt nanoscroll [48].

A number of efforts have been employed to increase the active sites and conductivity of TMDCs materials [106,107,108]. Among them, transforming the TMDCs nanosheets into nanoscrolls with active edges is of great interest in electrocatalytic HER [50]. By curling the TMDCs nanosheets to form nanoscrolls, the curled edges provide highly active edge sites for efficient catalysis. Meanwhile, the bending strain of the basal plane also provides more active sites due to the scrolled structure [50,109,110,111]. In addition, the specific surface area of the nanoscroll increases, converting the solution contact from single-sided contact of the nanosheet to multi-layer contact [112]. As a result, the TMDCs-NS provides higher catalytic activity and better conductivity. Thus, the HER activity of TMDCs-NS is greatly enhanced. Figure 13a shows the polarization curves of metallic WSe_2_ (M-WSe_2_) and 2H-WSe_2_ nanoscrolls compared to the commercial Pt/C catalyst. The M-WSe_2_ nanoscroll exhibits higher HER activity, smaller overpotential, and larger current densities than the 2H-WSe_2_ nanoscroll, attributed to its good conductivity and enhanced catalytic activity from the scrolled structure [103]. Figure 13b shows the linear scanning voltammogram curves of current density and potential for Pt electrode, MoS_2_ sheet, MoS_2_ nanoscroll, MoS_2_ nanoscrolls@Pt, MoS_2_@Pt sheet, and MoS_2_@Pt nanoscroll [48]. The MoS_2_ nanosheet shows overpotential over 400 mV, while that of the MoS_2_ nanoscroll decreases. The overpotential of MoS_2_ nanoscroll decorated by Pt nanoparticles (NPs) greatly decreases, indicating the important role of Pt NPs in enhancing the HER activity. By decorating Pt NPs on MoS_2_ nanosheet (MoS_2_@Pt sheet), the overpotential further decreases to around 300 mV. After the MoS_2_@Pt sheet was rolled up to form a MoS_2_@Pt scroll, the overpotential was reduced to 200 mV, implying the basal plane bending of the nanoscroll can further improve the HER activity. 

### 4.4. Gas Sensor 

In recent years, TMDCs nanosheets have attracted great attention for gas sensing because of their large surface-to-volume ratio and good electrical properties [113,114,115,116]. The TMDCs nanosheet-based gas sensors show high sensitivity for trace gas molecules and good selectivity at room temperature [60,117,118,119]. However, they suffer from incomplete recovery and poor stability [120], which limit their practical applications. The TMDCs-NS has good conductivity due to its 1D structure and specific surface area from the tubular structure. Meanwhile, the nanoscroll structure also provides tunable space to encapsulate functional nanomaterials for further enhanced performance. Therefore, the TMDCs-NS has been considered to present promising performance in the field of gas sensing [121]. 

**Figure 14:**
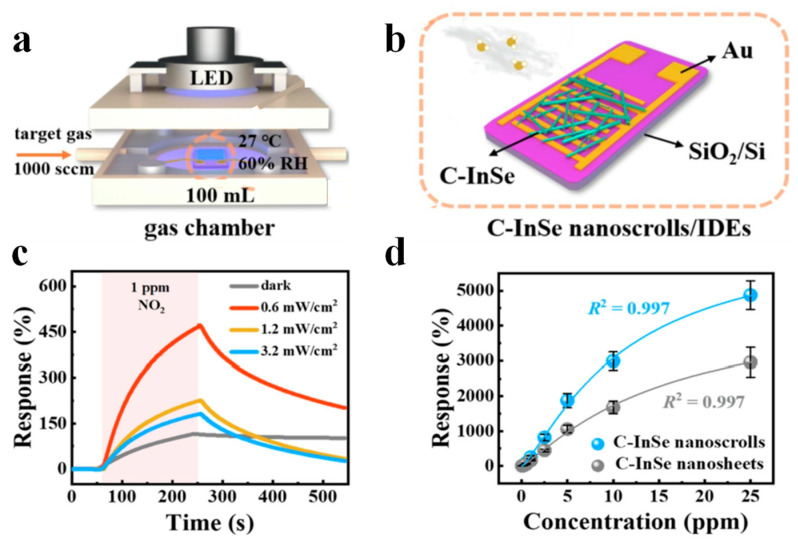
Gas sensor based on C-InSe nanoscrolls [120]. (**a**,**b**) Schematic illustration of C-InSe nanoscroll-based gas sensor and test platform. (**c**) Response curves of C-InSe nanoscrolls sensor towards 1 ppm NO_2_ under blue light irradiation with different light intensities. (**d**) Relationship between the response of C-InSe nanosheets and nanoscrolls based sensors and NO_2_ concentration.

By electrochemically exfoliating InSe crystal in electrolytes containing cetyltrimethylammonium bromide (CTAB), the CTAB-functionalized InSe nanosheets (C-InSe) were obtained [120]. With the aid of solvent evaporation, the C-InSe nanosheets rolled up to form C-InSe nanoscroll. After the as-obtained C-InSe nanoscrolls were deposited on interdigitated electrodes (Figure 14b), they were placed into a chamber with a fixed concentration of NO_2_ gas. An LED lamp illuminates light into the chamber through a glass cover (Figure 14a). The response of the C-InSe nanoscrolls-based sensor increases under light illumination (Figure 14c). The enhanced response has arisen from the increased adsorption of NO_2_ molecules favored by photogenerated electrons. While desorption of NO_2_ is observed as the light intensity increases further, which decreases the response of the sensor. As the concentration of NO_2_ increases from 100 ppb to 25 ppm, the response of C-InSe nanosheets and nanoscrolls-based sensors largely increases (Figure 14d). However, the C-InSe nanoscrolls-based sensor exhibits a much better response than the C-InSe nanosheets-based sensor, indicating the superiority of nanoscrolls in gas sensing.

## 5. Conclusions

**Figure 15:**
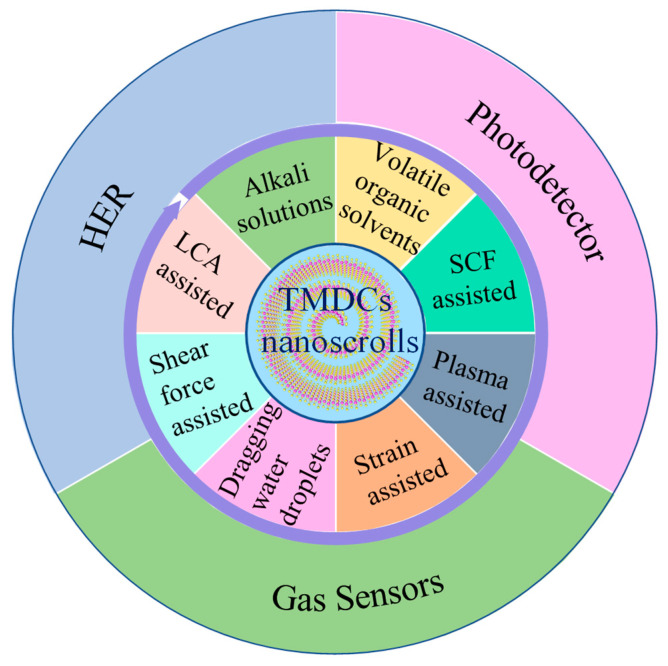
Summary of preparation and applications of TMDCs nanoscrolls.

In this review, we summarize a series of fabrication methods of TMDCs nanoscrolls, and briefly demonstrate their applications in photodetection, HER, and gas sensing (Figure 15). Compared to the 2D TMDCs nanosheet, the 1D TMDCs nanoscroll presents higher light absorption efficiency and faster electron transport because of the scrolled structure. Due to their higher specific surface area and active edges, the TMDCs nanoscrolls have shown excellent performance in catalytic reactions. In conclusion, the TMDCs nanoscrolls are emerging materials with many novel physical and chemical properties that are promising for optoelectronics, catalysis, energy storage, and sensing.

## Figures and Tables

**Table 1:** The advantages and disadvantages of preparation methods for TMDCs nanoscrolls.

Methods		Advantages	Challenges	References
In liquid	Solvent evaporation to make nanoscroll	Large area, large size, high productivity, short time consuming, easy to operate	Solvent residue, loose nanoscrolls	[22,23,24,25,33,53,56,67,70,79,97]
	Alkaline droplet-assisted fabrication of nanoscroll	High yield, high productivity for thick nanosheet	Substrate etching, solvent residue	[19,21,26,27,35,51]
	Fabrication of TMDCs nanoscroll by dragging water droplet	High yield, solvent-free residue, tightly packed nanoscroll	Not suitable for water and oxygen-sensitive material	[52]
	Amine-functional amphiphilic molecule assisted fabrication of TMDCs nanoscroll	High yield, easy to operate	Small dimension, solvent residue	[48,49,82]
	Supercritical fluid-assisted fabrication of nanoscroll	Simple, fast	Small dimension, solvent residue	[88]
	Shear force-assisted fabrication of nanoscroll	High productivity, easy to operate	Low proportion of monolayer nanosheet	[50]
In air	Plasma-assisted fabrication of TMDCs nanoscroll	Simple process, high yield	Small dimension, structural damage	[18,20]
	Strain-assisted fabrication of TMDCs nanoscroll	Simple and repeatable	Complex process, low productivity, incomplete nanoscroll	[17]

## Data Availability

Data can be available upon request from the authors.
